# Maternal Dietary Patterns during Pregnancy in Relation to Offspring Forearm Fractures: Prospective Study from the Danish National Birth Cohort

**DOI:** 10.3390/nu7042382

**Published:** 2015-04-02

**Authors:** Sesilje B. Petersen, Morten A. Rasmussen, Sjurdur F. Olsen, Peter Vestergaard, Christian Mølgaard, Thorhallur I. Halldorsson, Marin Strøm

**Affiliations:** 1 Centre for Fetal Programming, Department of Epidemiology Research, Statens Serum Institute, 2300 Copenhagen, Denmark; E-Mails: sfo@ssi.dk (S.F.O.); lur@ssi.dk (T.I.H.); mrm@ssi.dk (M.S.); 2 Department of Food Science, Faculty of Science, University of Copenhagen, 1958 Frederiksberg, Denmark; E-Mail: mortenr@food.ku.dk; 3 Copenhagen Prospective Studies on Asthma in Childhood, Copenhagen University Hospital, 2820 Gentofte, Denmark; 4 Department of Nutrition, Harvard School of Public Health, Boston, MA 02115, USA; 5 Department of Endocrinology, Aalborg University Hospital, 9000 Aalborg, Denmark; E-Mail: p-vest@post4.tele.dk; 6 Department of Clinical Medicine, Aalborg University, 9000 Aalborg, Denmark; 7 Department of Nutrition, Exercise and Sports, Faculty of Science, University of Copenhagen, 1958 Frederiksberg, Denmark; E-Mail: cm@nexs.ku.dk; 8 Faculty of Food Science and Nutrition, University of Iceland, 101 Reykjavik, Iceland

**Keywords:** maternal diet, dietary patterns, bone fractures, epidemiology, pregnancy, fetal programming, artificial sweetener

## Abstract

Limited evidence exists for an association between maternal diet during pregnancy and offspring bone health. In a prospective study, we examined the association between dietary patterns in mid-pregnancy and offspring forearm fractures. In total, 101,042 pregnancies were recruited to the Danish National Birth Cohort (DNBC) during 1996–2002. Maternal diet was collected by a food frequency questionnaire. Associations were analyzed between seven dietary patterns extracted by principal component analysis and offspring first occurrence of any forearm fracture diagnosis, extracted from the Danish National Patient Register, between time of birth and end of follow-up (<16 year) (*n* = 53,922). In multivariable Cox regression models, offspring of mothers in the fourth *vs.* first quintile of the Western pattern had a significant increased risk (Hazard ratio, 95% confidence interval: 1.11, 1.01–1.23) of fractures, and there was a borderline significant positive trend (*p* = 0.06). The other dietary patterns showed no associations and neither did supplementary analyses of macro- and micronutrients or single food groups, except for the intake of artificially sweetened soft drinks, which was positively associated with offspring forearm fractures (*p* = 0.02). In the large prospective DNBC high mid-pregnancy consumption of Western diet and artificially sweetened soft drinks, respectively, indicated positive associations with offspring forearm fractures, which provides interesting hypotheses for future research.

## 1. Introduction

It is well known that low bone mass in old age, which constitutes a major public health concern, can be prevented by optimal accumulation of bone mass during childhood and adolescence. The bone mass reaches a plateau (the peak bone mass) in the twenties, and from then it remains relatively constant until middle age, when it starts to decline and continues to do so throughout life [1]. The peak bone mass is influenced by lifestyle factors, such as diet and physical activity, during childhood and adolescence [2]. A growing body of evidence also suggests that adult bone mass may be influenced by factors operating as early as in fetal life [3].

The hypothesis of fetal programming of bone health has mainly been studied by analyzing the association between maternal Vitamin D status during pregnancy and offspring bone mineral content (BMC) and bone mineral density (BMD) measured by dual-energy X-ray absorptiometry (DXA) at a single time point during childhood, but the results are conflicting [4,5,6,7,8]. Few studies have explored the relation between maternal diet during pregnancy and offspring accumulation of bone mass measured by DXA during childhood [9,10,11,12,13,14,15,16]. There seems to be some evidence for a negative association with respect to dietary fat intake, but a positive association with respect to calcium, magnesium and folate with offspring BMD. However, in most of the studies, the potential associations with patterns in dietary intake were not considered and the analyses were limited to associations with single foods and nutrients. Thus, results may be prone to bias by correlated foods or nutrients that may potentiate or attenuate the effect of others.

In the large prospective Danish National Birth Cohort detailed data on dietary intake in mid-pregnancy was collected from more than 69,000 pregnant women [17]. At recruitment, the women provided consent for themselves and their unborn child for later data linkage to Danish health registers, which gives us a unique opportunity to examine the association between dietary patterns during pregnancy and offspring bone health, measured by incidence of forearm fractures. Pediatric fractures, and especially forearm fractures, seem to be a reliable predictor of child bone health, since a meta-analysis from 2006 indicated an association between low BMD and childhood bone fractures [18], and a review from 2010 found consistent and convincing evidence for an association between BMD and the risk of forearm fractures in childhood [19]. Therefore, the aim of the present study was in a prospective design to examine the association between maternal dietary patterns during pregnancy and offspring forearm fractures during childhood and adolescence in the Danish National Birth Cohort.

## 2. Materials and Methods

### 2.1. Study Population

The Danish National Birth Cohort (DNBC) recruited 101,042 pregnancies (103,145 mother and child-pairs) between 1996 and 2002 during the first antenatal visit to the general practitioner around weeks 6–10 of gestation. Approximately 35% of all pregnancies in Denmark in the recruitment period were included in the cohort, which has been described in detail elsewhere [17]. The original data collection included two telephone interviews during pregnancy in gestation weeks 12 and 30, two after delivery at 6 and 18 months postpartum, as well as a semi-quantitative food frequency questionnaire (FFQ) that was mailed to the women in gestation week 25 [17,20]. The DNBC complies with the Declaration of Helsinki and was approved by the Danish National Committee on Biomedical Research Ethics.

### 2.2. Exposure

#### 2.2.1. Assessment of Nutrient Intake

Information about maternal diet in pregnancy was available from the self-administered FFQ in mid-pregnancy, where the women were asked about their dietary intake in the previous four weeks. The semi-quantitative FFQ comprised questions on frequency of intake of approximately 360 different items of foods and beverages [20]. To quantify the total dietary consumption of food and beverages, standard portion sizes and standard recipes were applied for all items in the questionnaire. Standard portion sizes were multiplied with the daily frequencies to estimate intake of 65 food groups in grams [21] and then coupled with the Danish Food Tables to estimate nutrient intakes [21].

#### 2.2.2. Assessment of Dietary Patterns

Seven dietary patterns have previously been extracted by principal component analysis (PCA) on dietary information from 69,305 women in the DNBC [22]. Based on the food items with high factor correlations, the dietary patterns were named Prudent, Alcohol, Western, Seafood, Nordic, Sweets, and Rice/Pasta/Poultry (in this study named Traditional). Together these patterns explained 30.6% of the total variation in data. A short characterization of the seven dietary patterns is shown in Table 1. Detailed description of the method used for extracting the dietary patterns, and a further characterization of the patterns is given elsewhere [22].

### 2.3. Outcome

The outcome was defined as first occurrence of any forearm fracture diagnosis, extracted from the Danish National Patient Register (DNPR) by means of the unique Danish personal identifier (CPR) [23,24,25]. The diagnoses in the DNPR have been recorded since 1995 as the 10^th^ version of the International Classification of Diseases (ICD-10), and we used the following codes for forearm fractures in the study: DS525, DS526, DS525A, DS525B and DS525C. From the DNPR we also extracted variables registered along with the fracture diagnosis, such as time and date of accident, cause of accident, involvement in a traffic accident, time and date of discharge, *etc.* The DNPR is a mandatory nationwide register established in 1977 recording information from all hospital admissions, including outpatient activities and emergency room contacts [24]. The register has a high precision of diagnoses both in general [23], and for fracture diagnoses in particular [26].

**Table 1:** Short characterization of the seven dietary patterns in the Danish National Birth Cohort.

	Prudent	Alcohol	Western	Nordic	Shellfish	Sweets	Traditional
**High In**	Vegetables	Alcohol	Meat	Dark bread	Fish	White bread	Poultry
	Legumes	Soy	Potatoes	Nordic fruit	Shellfish	Cakes	Meat
	Root	Root	White bread	Cheese	Lamb	Margarine	Low-fat milk
	Fruit	Soft drinks	Egg	Banana	Oils	French fries	Meat
	Corn	Berries	Margarine	Cakes	Egg	Soft drinks, sugar	Water
**Low In**	Meat	Pasta/rice	Vegetables	French fries	Soft drinks, diet	Low-fat milk	Full fat milk
	French Fries	Yogurt	Fruit	Candy/snack	Candy/snack	Cabbage	Coffee
	Margarine	Poultry	Breakfast cereals	Soft drinks	Low-fat milk	Fruits	Butter
	White bread	Cheese	Nuts	Processed meat	Coffee	Fish	Potatoes
	Candy/snack	Bread	Water	Desserts	White bread	Legumes	White bread

### 2.4. Statistical Methods

**Figure 1:**
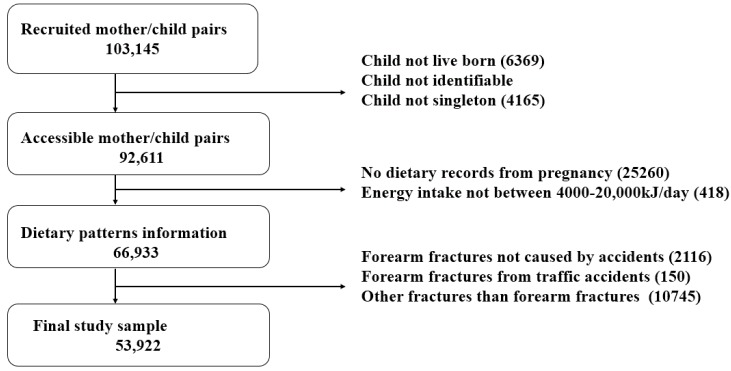
Flow chart of the steps in the derivation of the final study sample.

Children in the study sample were followed from the date of birth until the age of first forearm fracture, other censoring event, or the defined end of follow up, which was 10 November 2013. In total, we censored 1891 children based on data about emigration and death from the Danish Civil Registration System [25]. Our final study sample of 53,922 children was restricted to live births, singletons, offspring with a validated CPR number (Figure 1), and maternal energy intake between >4000 and <20,000 kJ day^−1^ (>956 and <4780 kcal day^−1^). Further, only fractures from accidents, excluding those caused by traffic accidents, which most likely are high-energy fractures, were included in the study. We also excluded offspring with a first time bone fracture in other areas than the forearm.

Associations between dietary patterns in pregnancy and offspring forearm fractures were analyzed using Cox proportional hazard models with age in days as the underlying time scale. Study participants were considered to be at risk for a forearm fracture from the time of birth until the age of first forearm fracture, other censoring event, or the defined end of follow up, whichever came first. We calculated hazard ratios (HRs) and 95% confidence intervals (CIs), and used Kaplan-Meier estimates to visualize the associations. The main analyses included the seven variables for dietary patterns: Prudent, Alcohol, Western, Seafood, Nordic, Sweets, and Traditional. The dietary patterns were analyzed both as quintiles and continuous values using the factor scores as a dietary exposure surrogate. Supplementary analyses followed an explorative strategy where we analyzed specific food groups and nutrients related to the patterns that showed significant associations with offspring forearm fractures. These supplementary analyses were conducted with the food item or nutrient as exposure with and without adjusting for relevant dietary patterns in order to reveal associations beyond general dietary habits. All analyses were carried out using SAS statistical software (version 9.4; SAS Institute, Cary, NC, USA).

The following covariates, which were defined by data extraction from either the enrollment form, the two telephone interviews or the FFQ were included in the study: Maternal age and pre-pregnancy body mass index (BMI in kg m^−2^) (both continuous), occupational status (unemployed, unskilled, student, skilled, medium, high), cohabitation status (couple, single), maternal smoking (non-smoker, occasional smoker, <15 cigarettes per day, ≥15 cigarettes per day), parity (no children, 1 child, 2 children, 3 + children), maternal physical activity in minutes per week (0, 1–44, 45–74, 75–149, ≥150), child sex (male, female), season of birth (winter (December/January/February), spring (March/April/May), summer (June/July/August), autumn (September/October/November)), gestational age (continuous) and birth weight (continuous). We substituted missing values of covariates by the median/mode method; the proportions of missing values were in the range from 0.004% (gestational age) to 11.5% (physical activity).

For supplementary analyses, we selected the following food groups for analysis based on the results from dietary patterns analyses, and based on previous knowledge of food items of potential relevance in childhood bone health: Alcohol, soy, margarine, meat, egg, white bread, French fries, candy, dairy products, fish, vegetables, coffee and soft drinks. Further, we selected the following macro and micronutrients for analyses: Protein, animal protein, vegetable protein, fat, carbohydrate, saturated fatty acids, *n*-3 and *n*-6 fatty acids, Vitamin A, B12, C, D, E, K, folate, calcium, magnesium, and phosphorus. Food groups and nutrients were both analyzed in quintiles and as continuous variables, except for French fries (categorized as quartiles), artificially sweetened (AS) soft drinks (categorized 0 g day^−1^, 0–10 g day^−1^, ≥10–60 g day^−1^, ≥60 g day^−1^) and soy (categorized 0 g day^−1^, 0–10 g day^−1^, ≥10 g day^−1^), because a high proportion of the study population reported zero intake of those food items. In analyses using continuous intakes, we excluded distinct outliers.

In sensitivity analyses, we stratified relevant analyses by pre-pregnancy BMI and offspring sex.

Furthermore, we used the following sub-categorization of soft drink intake: Carbonated sugar sweetened (SS) soft drinks, non-carbonated SS soft drinks, carbonated AS soft drinks, and non-carbonated AS soft drinks.

## 3. Results

In total, 53,922 mother and child-pairs were available for analysis (Figure 1) of which 4222 offspring were diagnosed with a first time forearm fracture during follow up. Mean age (SD) at first forearm fracture was 8.3 (3.4) in males and 7.9 (3.2) in females. Background characteristics of the mothers according to propensity of forearm fractures are shown in Table 2*.*

**Table 2:** Background characteristics of the study sample in the Danish National Birth Cohort according to fracture propensity in the offspring during childhood (*n* = 53,922).

	Forearm Fracture		
	Yes *n* = 4222	No *n* = 49,700	*p*
Maternal age (mean)	30.44	30.46	0.80 ^a^
Pre-pregnancy BMI, kg m^−2^ (mean)	23.66	23.46	0.002 ^a^
Occupational status (%)			
High	23.5	22.9	0.50 ^b^
Medium	34.6	35.0	
Skilled	26.1	25.7	
Student	4.5	5.1	
Unskilled	9.6	9.8	
Unemployed	1.6	1.5	
Cohabitation status (%)			
Single	1.56	1.67	0.60 ^b^
Couple	98.4	98.3	
Smoking (%)			
Non-smoker	76.5	76.1	0.83 ^b^
Occasional smoker	12.0	12.2	
<15 cigarettes/day	9.8	9.9	
≥15 cigarettes/day	1.6	1.8	
Parity (%)			
Nulliparous	48.6	51.7	<0.001 ^b^
1 child	37.0	34.0	
2 children	12.4	11.7	
3+ children	2.0	2.6	
Physical activity, minutes week^−1^ (%)			
0	54.3	55.2	0.011 ^b^
1–44	15.5	14.9	
45–74	10.9	11.0	
75–149	10.9	11.8	
≥150	8.3	7.1	
Child sex (%)			
Male	50.3	50.4	0.89 ^b^
Female	49.7	49.6	
Season of birth (%)			
Mar/Apr/May	23.3	23.4	0.023 ^b^
Jun/Jul/Aug	26.0	26.5	
Sep/Oct/Nov	28.5	26.5	
Dec/Jan/Feb	22.2	23.6	
Gestational age (mean)	280.5	280.2	0.15 ^a^
Birth weight (mean)	3624	3583	<0.001 ^a^

^a^ *p*-value from ANOVA for measure of association; ^b^ *p*-value from chi-square test for measure of association.

The Prudent, Nordic, Seafood and Traditional pattern were not associated with offspring forearm fractures, neither in crude analyses nor when we adjusted for potential confounders (Table 3).

The Alcohol and Sweet patterns showed borderline significant associations: For the Sweets pattern, the overall categorical test of association was borderline significant, and for the Alcohol pattern pairwise comparisons showed a borderline significant higher risk for the third *vs*. second quintile. However, for both patterns there were no trends when the variables were analyzed as continuous (Table 3). Stratifying by sex in the analysis of the Alcohol pattern revealed that the increased risk for the third *vs.* second quintile was confined to male offspring only (HR, 95% CI: 1.16, 1.01–1.34).

The overall test of association for the Western pattern did not indicate any association with forearm fractures, but pairwise comparisons showed that offspring of mothers in the fourth *vs.* first quintile had a significant increased risk, and offspring of mothers in the fifth *vs.* first quintile had borderline significant increased risk of forearm fractures (Table 3). There was also a borderline significant trend when the Western pattern was analyzed as a continuous variable (Table 3).

No significant associations were found in the separate analyses on the food groups related to the dietary patterns that indicated association with offspring forearm fractures (Table S1), except for AS soft drinks (Table 4). The Western pattern is among other dietary factors characterized by a high intake of meat, but the overall test of association for meat intake did not indicate any association with offspring forearm fractures. Pairwise comparisons for the meat intake showed that offspring had a borderline significant lower risk in third *vs.* fifth quintile, but no trend was found when the meat intake was analyzed continuously (Table 5). When we adjusted for meat in the Western pattern analysis, the association was strengthened, with offspring in fifth *vs.* first quintile having significant higher risk of forearm fractures (HR, 95% CI: 1.15, 1.02–1.30), and a significant positive trend of association (*p* = 0.03); thus a high intake of meat could not explain the association for Western pattern. Further analyses on different sources of protein indicated no significant associations between intakes of animal and vegetable protein, respectively, and offspring forearm fractures (Table 5).

Analyses on macro and micronutrients did not indicate associations between specific vitamins and minerals and offspring forearm fractures (Table S2). We found a significantly increased risk for second *vs.* first quintile for dietary Vitamin D, but no trend when entering Vitamin D into the model as a continuous variable (Table S2). Adjustment for dietary patterns had no relevant effect on the estimates (data not shown).

**Table 3:** Hazard ratios (HRs) of offspring forearm fractures in the Danish National Birth Cohort according to maternal dietary patterns in mid-pregnancy (*n* = 53,922).

		Cases	Crude HR (95% CI)	*p*	Adjusted HR (95% CI) *	*p*
Prudent pattern	Q1	860	1.00	0.81 ^a^	1.00	0.79 ^a^
	Q2	856	1.01 (0.92, 1.11)	0.98 ^b^	1.01 (0.91, 1.11)	0.87 ^b^
	Q3	859	1.02 (0.92, 1.12)		1.01 (0.92, 1.12)	
	Q4	820	0.97 (0.88, 1.07)		0.97 (0.88, 1.07)	
	Q5	857	1.03 (0.93, 1.13)		1.03 (0.93, 1.14)	
Alcohol pattern	Q1	823	1.00	0.05 ^a^	1.00	0.05 ^a^
	Q2	806	0.95 (0.86, 1.05)	0.86 ^b^	0.95 (0.86, 1.05)	0.79 ^b^
	Q3	917	1.10 (1.00, 1.20)		1.09 (0.99, 1.20)	
	Q4	861	1.03 (0.93, 1.13)		1.02 (0.93, 1.13)	
	Q5	845	0.99 (0.90, 1.09)		0.99 (0.90, 1.09)	
Western pattern	Q1	804	1.00	0.15 ^a^	1.00	0.20 ^a^
	Q2	841	1.04 (0.95, 1.15)	0.06 ^b^	1.03 (0.94, 1.14)	0.06 ^b^
	Q3	828	1.04 (0.94, 1.14)		1.03 (0.93, 1.14)	
	Q4	903	1.12 (1.02, 1.23)		1.11 (1.01, 1.23)	
	Q5	876	1.09 (0.99, 1.20)		1.09 (0.98, 1.21)	
Nordic pattern	Q1	844	1.00	0.50 ^a^	1.00	0.47 ^a^
	Q2	850	0.98 (0.89, 1.08)	0.63 ^b^	0.98 (0.89, 1.08)	0.59 ^b^
	Q3	808	0.94 (0.85, 1.03)		0.93 (0.85, 1.03)	
	Q4	880	1.02 (0.93, 1.12)		1.02 (0.92, 1.12)	
	Q5	870	0.99 (0.90, 1.09)		1.00 (0.90, 1.10)	
Seafood pattern	Q1	860	1.00	0.20 ^a^	1.00	0.19 ^a^
	Q2	803	0.94 (0.85, 1.03)	0.91 ^b^	0.94 (0.85, 1.03)	0.52 ^b^
	Q3	906	1.05 (0.95, 1.15)		1.05 (0.96, 1.16)	
	Q4	833	0.96 (0.87, 1.06)		0.97 (0.88, 1.08)	
	Q5	850	0.99 (0.90, 1.09)		1.01 (0.91, 1.12)	
Sweets pattern	Q1	866	1.00	0.06 ^a^	1.00	0.05 ^a^
	Q2	883	1.02 (0.93, 1.12)	0.79 ^b^	1.02 (0.93, 1.12)	0.98 ^b^
	Q3	814	0.94 (0.86, 1.04)		0.94 (0.86, 1.04)	
	Q4	793	0.92 (0.83, 1.01)		0.92 (0.84, 1.02)	
	Q5	896	1.04 (0.95, 1.14)		1.05 (0.96, 1.16)	
Traditional pattern	Q1	884	1.00	0.94 ^a^	1.00	0.85 ^a^
	Q2	876	1.01 (0.92, 1.11)	0.70 ^b^	1.00 (0.91, 1.09)	0.41 ^b^
	Q3	826	0.97 (0.88, 1.07)		0.95 (0.86, 1.05)	
	Q4	843	1.00 (0.91, 1.10)		0.98 (0.89, 1.08)	
	Q5	823	0.99 (0.90, 1.09)		0.97 (0.88, 1.07)	

* Adjusted for maternal age, parity, cohabitation status, pre-pregnancy BMI, occupational status, maternal smoking, physical activity in pregnancy, offspring sex, gestational age and birth weight. ^a^ *p*-value from categorical χ^2^ test of overall association; ^b^ *p*-value from test of linear trend with intakes of soft drinks as a continuous variable.

**Table 4:** Hazard ratios (HRs) of offspring forearm fractures in the Danish National Birth Cohort according to maternal intake of soft drinks in mid-pregnancy (*n* = 53,922).

Estimated intake	Crude HR (95% CI)	Adjusted HR (95% CI) *	Mutually Adj. HR (95% CI) **
Sugar sweetened soft drinks, grams per day			
Q1 (0–45)	1.00	1.00	1.00
Q2 (46–80)	0.92 (0.84, 1.02)	0.93 (0.84, 1.02)	1.08 (0.98, 1.19)
Q3 (81–149)	1.01 (0.92, 1.11)	1.01 (0.92, 1.11)	1.10 (1.00, 1.21)
Q4 (150–257)	0.98 (0.89, 1.08)	0.98 (0.89, 1.08)	1.07 (0.97, 1.18)
Q5 (258–4000)	0.96 (0.88, 1.06)	0.97 (0.88, 1.07)	1.05 (0.95, 1.16)
	*p =* 0.42 ^a^; *p =* 0.71 ^b^	*p =* 0.43 ^a^; *p =* 0.80 ^b^	*p =* 0.38 ^a^; *p =* 0.81 ^b^
Artificially sweetened soft drinks, grams per day			
0	1.00	1.00	1.00
>0–10	0.98 (0.87, 1.10)	0.98 (0.87, 1.11)	0.98 (0.87, 1.10)
≥10–60	1.06 (0.98, 1.15)	1.06 (0.98, 1.15)	1.06 (0.98, 1.15)
≥60	1.14 (1.05, 1.23)	1.12 (1.04, 1.21)	1.12 (1.03, 1.21)
	*p* = 0.005 ^a^; *p* = 0.02 ^b^	*p* = 0.02 ^a^; *p* = 0.08 ^b^	*p* = 0.03 ^a^; *p* = 0.08 ^b^
Carbonated sugar sweetened soft drinks, servings per week			
Never	1.00	1.00	1.00
<1	0.92 (0.84, 1.02)	0.93 (0.84, 1.02)	0.94 (0.86, 1.04)
1–6	0.96 (0.88, 1.05)	0.97 (0.89, 1.05)	0.97 (0.89, 1.07)
≥7	0.93 (0.82, 1.05)	0.93 (0.82, 1.06)	0.96 (0.84, 1.11)
	*p* = 0.39 ^a^; *p* = 0.38 ^b^	*p* = 0.44 ^a^; *p* = 0.48 ^b^	*p* = 0.34 ^a^; *p* = 0.72 ^b^
Carbonated artificially sweetened soft drinks, servings per week			
Never	1.00	1.00	1.00
<1	1.04 (0.95, 1.14)	1.03 (0.94, 1.14)	1.03 (0.94, 1.14)
1–6	1.10 (1.01, 1.19)	1.08 (1.00, 1.18)	1.07 (0.98, 1.17)
≥7	1.02 (0.87, 1.19)	0.99 (0.84, 1.16)	0.95 (0.80, 1.14)
	*p* = 0.15 ^a^; *p* = 0.09 ^b^	*p* =0.28 ^a^; *p* = 0.26 ^b^	*p* = 0.34 ^a^; *p* = 0.50 ^b^
Noncarbonated sugar sweetened soft drinks, servings per week			
Never	1.00	1.00	1.00
<1	0.91 (0.82, 1.00)	0.90 (0.82, 1.00)	0.93 (0.84, 1.02)
1–6	0.95 (0.88, 1.02)	0.94 (0.87, 1.02)	0.96 (0.89, 1.05)
≥7	0.98 (0.90, 1.06)	0.97 (0.89, 1.05)	1.00 (0.91, 1.09)
	*p* = 0.21 ^a^; *p* = 0.54 ^b^	*p* = 0.17 ^a^; *p* = 0.40 ^b^	*p* = 0.43 ^a^; *p* = 0.91 ^b^
Noncarbonated artificially sweetened soft drinks, servings per week			
Never	1.00	1.00	1.00
<1	0.96 (0.85, 1.08)	0.96 (0.85, 1.08)	0.95 (0.84, 1.08)
1–6	1.05 (0.96, 1.15)	1.05 (0.56, 1.15)	1.04 (0.95, 1.15)
≥7	1.13 (1.04, 1.24)	1.13 (1.03, 1.23)	1.11 (1.01, 1.23)
	*p* = 0.02 ^a^; *p* = 0.005 ^b^	*p* = 0.04 ^a^; *p* = 0.009 ^b^	*p* = 0.10 ^a^; *p* = 0.03 ^b^

* Adjusted for maternal age, parity, cohabitation status, pre-pregnancy BMI, occupational status, maternal smoking, physical activity in pregnancy, offspring sex, gestational age and birth weight; ** Additionally adjustment: the first two types in one analysis (soft drinks with sugar, soft drinks AS), and the last four types in one analysis (carbonated with sugar, carbonated AS, noncarbonated with sugar, noncarbonated AS);^a^ *p*-value from categorical χ^2^ test of overall association; ^b^ *p*-value from test of linear trend with intakes of soft drinks as a continuous variable.

**Table 5:** Hazard ratios (HRs) of offspring forearm fractures in the Danish National Birth Cohort according to maternal dietary intakes of meat and protein in mid-pregnancy (*n* = 53,922).

Estimated intake	Crude HR (95% CI)	*p*	Adjusted HR (95% CI) *	*p* *
Meat				
Q1	0.98 (0.89, 1.08)	0.35 ^a^	0.99 (0.90, 1.09)	0.33 ^a^
Q2	0.98 (0.94, 1.08)	0.97 ^b^	0.99 (0.90, 1.08)	0.89 ^b^
Q3	0.91 (0.89, 1.00)		0.91 (0.83, 1.00)	
Q4	0.98 (0.90, 1.08)		0.98 (0.89, 1.08)	
Q5	1.00		1.00	
Protein, total				
Q1	0.97 (0.88, 1.07)	0.70 ^a^	0.99 (0.90, 1.09)	0.70 ^a^
Q2	1.04 (0.94, 1.14)	0.49 ^b^	1.05 (0.95, 1.15)	0.75 ^b^
Q3	0.98 (0.89, 1.08)		0.99 (0.90, 1.08)	
Q4	0.99 (0.90, 1.09)		0.99 (0.90, 1.09)	
Q5	1.00		1.00	
Animal protein				
Q1	0.96 (0.88, 1.06)	0.67 ^a^	0.96 (0.88, 1.06)	0.67 ^a^
Q2	1.00 (0.91, 1.10)	0.34 ^b^	1.00 (0.91, 1.10)	0.41 ^b^
Q3	0.95 (0.86, 1.04)		0.95 (0.86, 1.04)	
Q4	0.96 (0.87, 1.05)		0.96 (0.87, 1.05)	
Q5	1.00		1.00	
Vegetable protein				
Q1	0.99 (0.90, 1.09)	0.62 ^a^	0.99 (0.90, 1.08)	0.58 ^a^
Q2	0.95 (0.86, 1.05)	0.97 ^b^	0.95 (0.86, 1.04)	0.87 ^b^
Q3	0.95 (0.86, 1.04)		0.95 (0.86, 1.04)	
Q4	0.94 (0.85, 1.03)		0.94 (0.85, 1.03)	
Q5	1.00		1.00	

* Adjusted for maternal age, parity, cohabitation status, pre-pregnancy BMI, occupational status, maternal smoking, physical activity in pregnancy, offspring sex, gestational age and birth weight. ^a^ *p*-Value from categorical χ^2^ test of overall association. ^b^ *p*-Value from test of linear trend with intakes of soft drinks as a continuous variable.

Offspring of mothers in the highest intake group of AS soft drinks had a significant increased risk for forearm fractures compared with children of mothers who reported no intake. This was also reflected when the intake was analyzed as a continuous variable (Table 4). Mutual adjustment with the two types of soft drinks still indicated significant increased risk for high intake of AS soft drinks (Table 4). Sensitivity analyses also indicated a significant association between AS soft drinks and offspring forearm fractures, with increased fracture risk associated with intake of ≥1 *vs.* zero servings per day of non-carbonated AS soft drinks. There was also a significant positive trend when the intake was analyzed as a continuous variable (Table 4). These associations persisted after mutual adjustment of all four types of soft drinks (Table 4).

**Figure 2:**
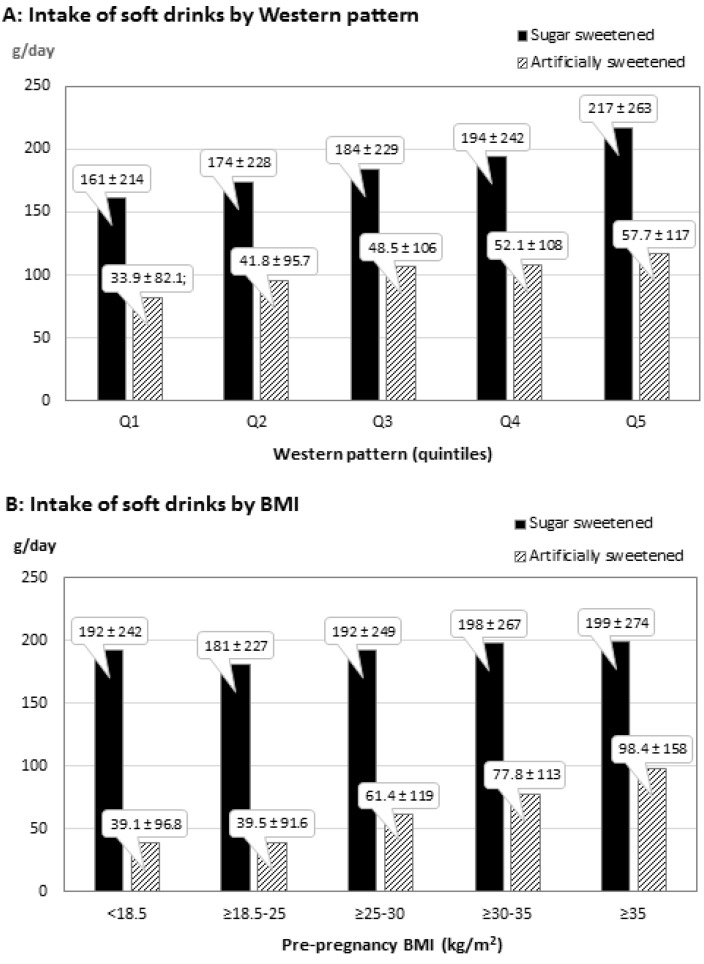
Maternal intakes in the Danish National Birth Cohort of sugar and artificially sweetened soft drinks (± SD) in mid-pregnancy (*n* = 53.922) according to Western dietary pattern and pre-pregnancy BMI.

In Figure 2 is shown the mean values for intake of soft drinks for quintiles of Western pattern (Figure 2A), and the mean intake stratified by BMI groups (Figure 2B).

We found no interaction between BMI and intake of AS soft drinks in the analysis, even though the mean intake of AS soft drinks was almost 150% higher among women with BMI ≥35 kg m^−2^ compared with ≥18.5–25 kg m^−2^. Stratifying by BMI revealed that AS soft drinks also was associated with offspring forearm fractures among women with a normal range BMI (≥18.5–25 kg m^−2^) for the highest intake group *vs.* zero intake (HR, 95% CI: 1.13, 1.02–1.25).

Adjustment for intake of AS soft drinks in the analyses of Western pattern did not attenuate the association markedly (HR, 95% CI: 1.11, 1.00–1.22, *p* = 0.06 for trend) as would be expected if AS soft drinks were the underlying food item of the association for Western pattern with offspring forearm fractures.

## 4. Discussion

In a study of 59,522 women from the DNBC we found overall limited evidence to suggest that dietary habits in mid-pregnancy may influence offspring later risk of forearm fractures in childhood. Still, our results did indicate that the Western pattern, characterized by high intake of fat, meat and potatoes, and low intake of fruit and vegetables, was associated with offspring forearm fractures. Secondary analyses with the aim to explore which components might explain the association with the Western pattern indicated that a high AS soft drink consumption modestly increased the risk of offspring forearm fractures (~12%). The intake of AS soft drinks could not explain the association with the Western pattern even though the two were closely correlated (mothers in the highest quintile of the Western pattern had a mean intake of AS soft drinks that was 70% higher than in the lowest quintile). This may suggest that if there truly is an association, it may be due to clustering of several weak risk factors that individually do not reach significance when explored one by one.

### 4.1. Western Diet in Relation to Bone Health

Previous studies have found an association between high consumption of Western type diet in women and low BMD [27,28]. This association has been thought to be due to high consumption of animal protein and low consumption of vegetable protein in the Western diet, because of higher urinary calcium excretion (each 10 g of protein increases urinary excretion by 16 mg), but the results in the field are conflicting [29,30]. In our study we found no associations between the intakes of total, animal, or vegetable protein and offspring forearm fractures. In fact, we found a strengthened association between Western pattern and offspring forearm fractures when we adjusted for meat intake, indicating that animal protein may even be protective if anything. We also found no associations between single nutrients and offspring forearm fractures, which indicated that the modest association for maternal Western diet with offspring forearm fracture risk might be due to factors other than the total maternal intake of nutrients during pregnancy. For example, concerns have been expressed about the possible adverse effects of the low calcium to phosphorus ratio and the high ratios between the different types of fatty acids in Western typed diet [31,32,33]. We did not go further into analyzing nutrient ratios, but this might be relevant to address in further studies.

In relation to osteoporosis, there has been much debate on the acid ash hypothesis [34,35] that assumes that even mild, nonclinical acidotic changes in the physiological pH adversely affect bone mass [30]. There is concern that with the typical Western diet, the body is permanently in a state of net endogenous acid production that increases urinary acid and calcium excretion [35,36], because of high consumption of acid producing food items when metabolized (e.g., animal products and cereals) and low consumption of alkaline producing food items (e.g., fruit and vegetables) [36]. Thus, high consumption of a Western type diet may in the long term generate a state of mild metabolic acidosis [36]. Animal and *in vitro* studies support that an acid producing diet in the long-term adversely affects skeletal bone [34,35], but the studies in adult humans have shown conflicting results [34,35,37]. The acid ash hypothesis has not yet been addressed in relation to pregnancy, but it is speculative that also mild acidosis during pregnancy may negatively affect fetal bone development, because of the increased need for calcium during pregnancy.

### 4.2. Our Results in Relation to Existing Knowledge

Few studies have previously investigated the association between maternal diet in pregnancy and offspring bone health [9,10,11,12,13,14,15,16], and only one of them has examined the association between maternal dietary patterns and measures of offspring bone health. In the Princess Anne Cohort Study they found that high consumption of a prudent diet (healthy eating), characterized by high intake of fruit, vegetables, whole meal bread, rice, pasta, yoghurt and breakfast cereals, during late pregnancy was associated with greater bone size and BMD in 198 children at nine years of age [15]. Our study partly supports their findings as our Western pattern in most aspects corresponds to the inverse of the prudent pattern.

In animal studies, alcohol exposure during pregnancy has been found to impact offspring skeletal development in a non-beneficial manner [38,39]; however, our results did not support that finding. There was a tendency towards an association between the Alcohol pattern and offspring forearm fractures in our data, but this was only for males, and no significant association was found when the alcohol intake was analyzed separately in supplementary analysis. As expected, the consumption of alcohol among women in the DNBC was low with a mean (SD) intake of 22.6 (32.5) g day^−1^, which corresponds to approximately one glass of wine per week. Thirty percent of the mothers reported zero intake of alcohol in mid-pregnancy. Low intake of alcohol among the women in the DNBC may explain why our results do not support previous findings from animal studies.

In supplementary analyses, we found that a high intake of AS soft drinks, but not SS soft drinks was positively associated with offspring forearm fractures. Previous studies have found that a high intake of SS soft drinks during childhood decreases bone mass accrual [40,41] and increases bone fracture risk [42,43]. However, no study has yet addressed whether this also is the case for AS soft drinks. High intake of AS soft drinks may be a predictor for low intake of milk products, but since we found no association for intake of SS soft drinks and no association for intake of milk products, we do not expect this to be the underlying explanation. Another possible explanation for the association between high consumption of AS soft drinks and fractures may be that women with a high BMI or women with gestational diabetes choose AS soft drinks rather than SS soft drinks in order to minimize weight gain during pregnancy. Both maternal obesity and maternal diabetes in pregnancy are found to lower offspring bone mass [44,45,46]. We did not adjust the analyses for gestational diabetes, but we did include pre-pregnancy BMI in our multivariable regression models. We also stratified by pre-pregnancy BMI and found that AS soft drinks also significantly increased the risk of offspring forearm fractures among women with a BMI in the normal range, indicating that different soft drink choices across BMI span was not the underlying cause for the association between maternal intake of AS soft drinks and forearm fractures in our data.

Sensitivity analyses indicated that the association with AS soft drinks relied primarily on the intake of non-carbonated types. In general, the content of artificial sweeteners differs between carbonated and non-carbonated soft drinks, with the first type often containing aspartame, while the sweeteners most often used in non-carbonated soft drinks are cyclamate and saccharine [47]. For decades the safety of cyclamate and saccharine have been widely studied in relation to any potential carcinogenic effects of the substances [48]. In an old animal study from 1979, saccharine added to the diet of weanling male rats was found to increase urinary excretion of calcium, magnesium and phosphorous (dose-related) and lower the urinary pH [49], which indicates that saccharine may affect the mineral balance in the body negatively. However, we can only speculate about this, since the safety of these types of sweeteners has not yet been investigated in relation to bone health.

In spite of our results, which were indicative of a modest relation between the Western type diet and offspring forearm fractures, we were not able to substantiate similar associations for any food items or nutrients, apart from AS soft drinks. In this regard, our results stand somewhat in contrast to previous studies that have investigated the association between maternal intake of single food items and nutrients during pregnancy in relation to DXA derived measures of bone mass in the offspring [9,10,11,12,13,14]. In general, the studies have been small and only one has followed the offspring beyond the first ten years of life [8]. Overall, there is some evidence for higher bone mass in the offspring if the mother ingested a diet low in total fat, but high in folate, magnesium and calcium-rich foods during pregnancy. Our study lends little support to these findings, possibly due to our endpoint being bone fractures, which is more complex and only an indirect predictor of BMD. However, bone fractures is a more functional measure of bone health and may better reflect the clinical relevance compared with DXA derived measures.

### 4.3. Strengths and Limitations

There are several strengths to our study, including the large study sample and high quality dietary assessment in mid-pregnancy. The FFQ has previously been validated both in a group of younger non-pregnant women [50], and in a subsample from the DNBC by a seven day weighed food diary and by biomarkers for the intake of fruit, vegetables, folate, protein, retinol and *n*-3 fatty acids [51,52]. Furthermore, for our outcome measure, we used data from the DNPR on forearm fractures, a measure that has previously been reported to have a high validity [25,26]. The dietary patterns in our study were extracted by PCA, which is a well-established and commonly used data driven statistical technique that produces new variables that are uncorrelated linear combinations of the dietary variables [53]. Analyzing individual nutrients in traditional analyses may potentiate or attenuate the effect of others, because many of the dietary constituents are collinear. Thus, analyzing dietary patterns instead of single foods and nutrients may give a more realistic measure of the total dietary intake.

One major limitation of our study is the lack of information about offspring lifestyle, weight and height during childhood. The peak bone mass is to a certain extent determined by heritability, but also physical activity [54,55], consumption of soft drinks and coffee have been associated with low bone mass, whereas milk products have been associated with high bone mass in adolescents [54,55]. Furthermore, obesity and risky behavior are related to bone fracture risks [56]; all factors that are not included in our analyses. Further, a limitation is the explorative strategy used for analysis of single food groups, micro- and macronutrients, which might increase the risk for type 1 errors by multiple testing. While explorative studies are appropriate for hypothesis generating studies one must be cautious when interpreting findings in an exposure-disease approach. Dietary patterns are population specific and depend upon geographical, cultural, and methodological variations, which complicates comparison between studies using PCA. The two most common names for dietary patterns extracted by PCA are Prudent and Western diet. However, the characterization of Prudent and Western pattern differ between studies and although similar nomenclature may be used, the patterns are not necessarily identical between study populations [57].

## 5. Conclusions

In conclusion, our study found little evidence that maternal diet may be an important determinant for offspring forearm fracture risk during childhood. There were indications that maternal Western diet was associated with offspring forearm fractures, and secondary analyses revealed that maternal consumption of AS soft drinks might be associated with offspring forearm fractures independently of the dietary pattern. However, it was not possible for us to identify any single food item in the Western pattern that appeared to be of importance for offspring forearm fracture risk. The increase in fracture risk with increased intake of these parameters was minor and confounding due to maternal weight gain or offspring postnatal lifestyle and behavior cannot be excluded. The clinical relevance of a lower maternal intake of Western diet and AS soft drinks during pregnancy can be discussed, since the difference in fracture risk was quite modest, approximately 10%–12% for the highest *vs.* lowest quintile of consumption. However, if 10% of the annual fractures can be prevented, that would be approximately 1000 pediatric fractures per year, just in Denmark, which is of high clinical relevance overall.

## Supplementary Files

Supplementary File 1
