# Supplementary material for: Maternal Dietary Patterns during Pregnancy in Relation to Offspring Forearm Fractures: Prospective Study from the Danish National Birth Cohort
Source: Nutrients. 2015 Apr 2;7(4):2382–400. doi: 10.3390/nu7042382 (PMC4425150; doi:10.3390/nu7042382)
Supplement: Supplementary File 1 [file nutrients-07-02382-s001.docx]

**Supplementary information**

**Table S1.** Hazard ratios (HRs) of offspring forearm fractures in the Danish National Birth Cohort according to maternal food intake in mid-pregnancy (*n* = 53,922).

| **Food group** | **Crude  HR (95% CI)** | **Adjusted  HR (95% CI) *** | **Food group** | **Crude  HR (95% CI)** | **Adjusted  HR (95% CI) *** |
| --- | --- | --- | --- | --- | --- |
| Egg |  |  | Dairy products |  |  |
| Q1 | 1.00 | 1.00 | Q1 | 1.00 | 1.00 |
| Q2 | 1.01 (0.92, 1.11) | 1.00 (0.91, 1.11) | Q2 | 1.02 (0.93, 1.12) | 1.02 (0.92, 1.12) |
| Q3 | 0.96 (0.88, 1.06) | 0.96 (0.88, 1.06) | Q3 | 1.00 (0.91, 1.11) | 1.00 (0.91, 1.10) |
| Q4 | 1.04 (0.95, 1.15) | 1.05 (0.95, 1.15) | Q4 | 1.02 (0.93, 1.13) | 1.02 (0.93, 1.12) |
| Q5 | 0.97 (0.88, 1.07) | 0.98 (0.89, 1.08) | Q5 | 1.00 (0.90, 1.10) | 0.99 (0.90, 1.09) |
|  | *p* = 0.52 ^a^ | *p* = 0.51 ^a^ |  | *p* = 0.97 ^a^ | *p* = 0.97 ^a^ |
|  | *p* = 0.81 ^b^ | *p* = 0.62 ^b^ |  | *p* = 0.79 ^b^ | *p* = 0.89 ^b^ |
| White bread |  |  | Margarine |  |  |
| Q1 | 1.00 | 1.00 | Q1 | 1.00 | 1.00 |
| Q2 | 0.97 (0.88, 1.07) | 0.97 (0.88, 1.07) | Q2 | 0.96 (0.87, 1.05) | 0.95 (0.86, 1.05) |
| Q3 | 1.00 (0.91, 1.11) | 1.00 (0.91, 1.10) | Q3 | 0.96 (0.87, 1.05) | 0.95 (0.86, 1.05) |
| Q4 | 1.06 (0.96, 1.16) | 1.05 (0.96, 1.16) | Q4 | 0.94 (0.86, 1.04) | 0.94 (0.85, 1.03) |
| Q5 | 1.04 (0.94, 1.14) | 1.04 (0.94, 1.14) | Q5 | 0.97 (0.88, 1.07) | 0.98 (0.89, 1.08) |
|  | *p* = 0.46 ^a^ | *p* = 0.41 ^a^ |  | *p* = 0.78 ^a^ | *p* = 0.70 ^a^ |
|  | *p* = 0.73 ^b^ | *p* = 0.63 ^b^ |  | *p* = 0.96 ^b^ | *p* = 0.78 ^b^ |
| Vegetables |  |  | Candy |  |  |
| Q1 | 1.00 | 1.00 | Q1 | 1.00 | 1.00 |
| Q2 | 0.99 (0.90, 1.09) | 0.99 (0.90, 1.09) | Q2 | 0.97 (0.88, 1.07) | 0.97 (0.88, 1.07) |
| Q3 | 0.98 (0.89, 1.08) | 0.98 (0.89, 1.08) | Q3 | 0.94 (0.86, 1.04) | 0.95 (0.86, 1.04) |
| Q4 | 0.94 (0.85, 1.03) | 0.94 (0.85, 1.04) | Q4 | 0.96 (0.87, 1.05) | 0.96 (0.87, 1.05) |
| Q5 | 1.02 (0.92, 1.12) | 1.02 (0.92, 1.12) | Q5 | 0.96 (0.87, 1.06) | 0.96 (0.87, 1.06) |
|  | *p* = 0.60 ^a^ | *p* = 0.59 ^a^ |  | *p* = 0.82^a^ | *p* = 0.84 ^a^ |
|  | *p* = 0.91 ^b^ | *p* = 0.99 ^b^ |  | *p* = 0.82^b^ | *p* = 0.93 ^b^ |
| Fish |  |  | Alcohol |  |  |
| Q1 | 1.00 | 1.00 | Q1 | 1.00 | 1.00 |
| Q2 | 0.99 (0.90, 1.09) | 0.99 (0.90, 1.09) | Q2 | 0.98 (0.87, 1.10) | 0.98 (0.87, 1.09) |
| Q3 | 0.98 (0.89, 1.08) | 0.98 (0.89, 1.08) | Q3 | 1.04 (0.95, 1.13) | 1.04 (0.96, 1.14) |
| Q4 | 0.98 (0.89, 1.08) | 0.98 (0.89, 1.08) | Q4 | 1.03 (0.94, 1.13) | 1.04 (0.95, 1.13) |
| Q5 | 1.03 (0.94, 1.14) | 1.03 (0.94, 1.14) | Q5 | 1.05 (0.96, 1.14) | 1.06 (0.97, 1.16) |
|  | *p* = 0.83 ^a^ | *p* = 0.82 ^a^ |  | *p* = 0.69 ^a^ | *p* = 0.57 ^a^ |
|  | *p* = 0.17 ^b^ | *p* = 0.14 ^b^ |  | *p* = 0.80 ^b^ | *p* = 0.62 ^b^ |
| French frites |  |  | Coffee |  |  |
| Q1 | 1.00 | 1.00 | Q1 | 1.00 | 1.00 |
| Q2 | 1.04 (0.95, 1.12) | 1.03 (0.94, 1.11) | Q2 | 1.00 (0.91, 1.09) | 1.01 (0.92, 1.10) |
| Q4 | 1.02 (0.94, 1.11) | 1.01 (0.93, 1.09) | Q3 | 0.95 (0.87, 1.03) | 0.95 (0.87, 1.04) |
| Q5 | 1.04 (0.93, 1.17) | 1.03 (0.91, 1.16) | Q4 | 0.95 (0.88, 1.08) | 0.96 (0.88, 1.05) |
|  | *p* = 0.83 ^a^ | *p* = 0.92 ^a^ |  | *p* = 0.45 ^a^ | *p* = 0.55 ^a^ |
|  | *p* = 0.51 ^b^ | *p* = 0.72 ^b^ |  | *p* = 0.41 ^b^ | *p* = 0.48 ^b^ |

**Table S1.** *Cont.*

| Soya |  |  |  |  |  |
| --- | --- | --- | --- | --- | --- |
| 0 g day^−1^ | 1.00 | 1.00 |  |  |  |
| 0–10 g day^−1^ | 0.92 (0.79, 1.07) | 0.93 (0.80, 1.08) |  |  |  |
| 0–10 g day^−1^ | 0.92 (0.79, 1.07) | 0.93 (0.80, 1.08) |  |  |  |
|  | *p* = 0.54 ^a^ | *p* = 0.64 ^a^ |  |  |  |
|  | *p* = 0.84 ^b^ | *p* = 0.76 ^b^ |  |  |  |

* Adjusted for maternal age, parity, cohabitation status, pre-pregnancy BMI, occupational status, maternal smoking, physical activity in pregnancy, offspring sex, gestational age and birth weight; a, Overall χ^2^ measure of association;
b, Continuous intakes of soft drinks in the Cox regression model.

**Table S2.** Hazard ratios (HRs) of offspring forearm fractures in the Danish National Birth Cohort according to maternal nutrient intake in mid-pregnancy (*n* = 54,240).

| **Nutrient *** | **Crude  HR (95% CI)** | **Adjusted  HR (95% CI) ^#^** | **Nutrient** | **Crude  HR (95% CI)** | **Adjusted  HR (95% CI) ^#^** |
| --- | --- | --- | --- | --- | --- |
| Carbohydrate |  |  | Vitamin C |  |  |
| Q1 | 1.00 | 1.00 | Q1 | 1.00 | 1.00 |
| Q2 | 0.97 (0.88, 1.06) | 0.95 (0.87, 1.05) | Q2 | 0.97 (0.88, 1.07) | 0.98 (0.87, 1.10) |
| Q3 | 0.92 (0.83, 1.01) | 0.91 (0.82, 1.00) | Q3 | 0.95 (0.87, 1.05) | 1.00 (0.90, 1.13) |
| Q4 | 0.95 (0.86, 1.04) | 0.93 (0.85, 1.03) | Q4 | 0.99 (0.91, 1.09) | 1.01 (0.90, 1.13) |
| Q5 | 0.99 (0.90, 1.09) | 0.98 (0.89, 1.08) | Q5 | 0.95 (0.86, 1.05) | 0.99 (0.88, 1.12) |
|  | *p* = 0.53 ^a^ | *p* = 0.38 ^a^ |  | *p* = 0.53 ^a^ | *p* = 0.61 ^a^ |
| Fat, total |  |  | Vitamin D |  |  |
| Q1 | 1.00 | 1.00 | Q1 | 1.00 | 1.00 |
| Q2 | 0.99 (0.90, 1.09) | 0.99 (0.90, 1.09) | Q2 | 1.11 (1.01, 1.22) | 1.16 (1.04, 1.30) |
| Q3 | 0.97 (0.88, 1.06) | 0.96 (0.88, 1.06) | Q3 | 1.00 (0.91, 1.10) | 1.01 (0.89, 1.13) |
| Q4 | 1.00 (0.91, 1.10) | 1.00 (0.91, 1.10) | Q4 | 1.11 (1.01, 1.22) | 1.08 (0.96, 1.21) |
| Q5 | 1.00 (0.91, 1.10) | 1.01 (0.92, 1.12) | Q5 | 1.05 (0.95, 1.16) | 1.08 (0.96, 1.21) |
|  | *p* = 0.88 ^a^ | *p* = 0.65 ^a^ |  | *p* = 0.75 ^a^ | *p* = 0.75 ^a^ |
| Saturated fat |  |  | Vitamin E |  |  |
| Q1 | 1.00 | 1.00 | Q1 | 1.00 | 1.00 |
| Q2 | 1.05 (0.95, 1.15) | 1.05 (0.95, 1.15) | Q2 | 0.99 (0.90, 1.09) | 0.99 (0.90, 1.09) |
| Q3 | 1.01 (0.91, 1.11) | 1.01 (0.91, 1.11) | Q3 | 1.08 (0.99, 1.19) | 1.09 (0.99, 1.20) |
| Q4 | 1.04 (0.94, 1.14) | 1.04 (0.94, 1.15) | Q4 | 1.06 (0.97, 1.17) | 1.08 (0.98, 1.19) |
| Q5 | 1.03 (0.94, 1.13) | 1.04 (0.95, 1.15) | Q5 | 1.02 (0.92, 1.12) | 1.04 (0.94, 1.15) |
|  | *p* = 0.65 ^a^ | *p* = 0.45 ^a^ |  | *p* = 0.61^a^ | *p* = 0.52 ^a^ |
| *n*-3 fatty acids |  |  | Vitamin K |  |  |
| Q1 | 1.00 | 1.00 | Q1 | 1.00 | 1.00 |
| Q2 | 1.09 (0.99, 1.20) | 1.09 (0.99, 1.20) | Q2 | 1.13 (1.03, 1.24) | 1.08 (0.96, 1.22) |
| Q3 | 1.04 (0.95, 1.15) | 1.04 (0.95, 1.15) | Q3 | 1.08 (0.98, 1.19) | 1.06 (0.95, 1.19) |
| Q4 | 1.09 (0.99, 1.20) | 1.09 (0.99, 1.20) | Q4 | 1.14 (1.04, 1.26) | 1.10 (0.98, 1.23) |
| Q5 | 1.06 (0.96, 1.17) | 1.07 (0.97, 1.18) | Q5 | 1.08 (0.98, 1.20) | 1.05 (0.93, 1.18) |
|  | *p* = 0.72 ^a^ | *p* = 0.51 ^a^ |  | *p* = 0.21 ^a^ | *p* = 0.74 ^a^ |

**Table S2.** *Cont.*

| *n*-6 fatty acids |  |  | Folate |  |  |
| --- | --- | --- | --- | --- | --- |
| Q1 | 1.00 | 1.00 | Q1 | 1.00 | 1.00 |
| Q2 | 1.05 (0.95, 1.15) | 1.05 (0.95, 1.15) | Q2 | 1.07 (0.97, 1.17) | 1.04 (0.93, 1.16) |
| Q3 | 1.06 (0.96, 1.17) | 1.06 (0.96, 1.17) | Q3 | 1.04 (0.94, 1.14) | 1.01 (0.90, 1.14) |
| Q4 | 1.10 (0.99, 1.21) | 1.09 (0.99, 1.20) | Q4 | 1.08 (0.98, 1.19) | 1.06 (0.94, 1.19) |
| Q5 | 1.10 (1.00, 1.21) | 1.10 (0.99, 1.21) | Q5 | 1.05 (0.95, 1.15) | 1.03 (0.92, 1.16) |
|  | *p* = 0.18 ^a^ | *p* = 0.23 ^a^ |  | *p* = 0.43 ^a^ | *p* = 0.62 ^a^ |
| *n*-3:*n*-6 ratio |  |  | Calcium |  |  |
| Q1 | 1.00 | 1.00 | Q1 | 1.00 | 1.00 |
| Q2 | 1.11 (1.01, 1.22) | 1.11 (1.01, 1.22) | Q2 | 0.97 (0.88, 1.06) | 0.97 (0.86, 1.09) |
| Q3 | 1.07 (0.97, 1.17) | 1.07 (0.97, 1.18) | Q3 | 1.00 (0.91, 1.10) | 0.98 (0.88, 1.10) |
| Q4 | 1.05 (0.96, 1.16) | 1.06 (0.96, 1.17) | Q4 | 0.96 (0.87, 1.06) | 0.96 (0.85, 1.07) |
| Q5 | 1.02 (0.92, 1.12) | 1.03 (0.93, 1.14) | Q5 | 0.98 (0.90, 1.08) | 0.97 (0.86, 1.09) |
|  | *p* = 0.35 ^a^ | *p* = 0.54 ^a^ |  | *p* = 0.83 ^a^ | *p* = 0.95 ^a^ |
| Vitamin A |  |  | Magnesium |  |  |
| Q1 | 1.00 | 1.00 | Q1 | 1.00 | 1.00 |
| Q2 | 1.00 (0.91, 1.10) | 0.98 (0.88, 1.10) | Q2 | 1.05 (0.95, 1.15) | 1.03 (0.92, 1.16) |
| Q3 | 0.95 (0.87, 1.05) | 0.94 (0.83, 1.05) | Q3 | 1.00 (0.90, 1.09) | 0.99 (0.88, 1.11) |
| Q4 | 1.00 (0.91, 1.10) | 1.03 (0.92, 1.15) | Q4 | 0.95 (0.86, 1.04) | 0.94 (0.84, 1.06) |
| Q5 | 0.99 (0.90, 1.09) | 0.97 (0.86, 1.09) | Q5 | 1.03 (0.93, 1.13) | 1.01 (0.90, 1.14) |
|  | *p* = 0.66 ^a^ | *p* = 0.28 ^a^ |  | *p* = 0.61 ^a^ | *p* = 0.36 ^a^ |
| Vitamin B12 |  |  | Phosphorus |  |  |
| Q1 | 1.00 | 1.00 | Q1 | 1.00 | 1.00 |
| Q2 | 1.01 (0.92, 1.11) | 1.02 (0.91, 1.14) | Q2 | 1.03 (0.94, 1.13) | 0.98 (0.88, 1.10) |
| Q3 | 1.07 (0.97, 1.17) | 1.12 (1.00, 1.25) | Q3 | 0.99 (0.90, 1.09) | 0.99 (0.88, 1.10) |
| Q4 | 0.97 (0.88, 1.06) | 0.97 (0.86, 1.09) | Q4 | 1.01 (0.92, 1.11) | 0.95 (0.85, 1.07) |
| Q5 | 1.05 (0.95, 1.15) | 1.03 (0.92, 1.16) | Q5 | 0.98 (0.89, 1.08) | 0.96 (0.86, 1.08) |
|  | *p* = 0.37 ^a^ | *p* = 0.77 ^a^ |  | *p* = 0.80 ^a^ | *p* = 0.70 ^a^ |

***** All estimated nutrient intakes were energy adjusted by the residual method described by Willett *et al*. [1]. ^#^ Adjusted for maternal age, parity, cohabitation status, pre-pregnancy BMI, occupational status, maternal smoking, physical activity
in pregnancy, offspring sex, gestational age and birth weight; a, Continuous values of dietary patterns in the Cox
regression model.

**Reference**

1. Willett, W.C.; Howe, G.R.; Kushi, L.H. Adjustment for total energy intake in epidemiologic studies. *Am. J. Clin. Nutr.* **1997**, *65*, 1220S–1228S.

© 2015 by the authors; licensee MDPI, Basel, Switzerland. This article is an open access article distributed under the terms and conditions of the Creative Commons Attribution license (http://creativecommons.org/licenses/by/4.0/).
